# Mismatched and underrepresented: bat–virus diversity and phylogeographic bias in experimental infection studies

**DOI:** 10.3389/fimmu.2026.1901431

**Published:** 2026-09-14

**Authors:** Luis Víquez-R, Thomas P. Mazzarulli, Melissa T. R. Hawkins, Ashlyn P. Sak, José F. González-Maya, Kenneth A. Field, DeeAnn M. Reeder

**Affiliations:** 1 Department of Biology, Bucknell University, Lewisburg, PA, United States; 2 National Museum of Natural History, Smithsonian Institution, Washington, DC, United States; 3 Universidad Autónoma Metropolitana-Lerma, Lerma, Mexico

**Keywords:** bats, bat-virus relationships, cell lines, Chiroptera, experimental infection, organoids, viral infection, zoonoses

## Abstract

Despite growing interest in bat–virus interactions, experimental infection studies (EIS) remain taxonomically and geographically restricted, limiting inference about bat immunology and zoonotic risk. We performed a systematic literature search (Jan 1900–Jan 2025) and manually curated peer-reviewed primary studies of viral experimental infections in live bats and bat-derived cell lines (BdCL). From 545 candidate records we retained 127 papers (83 live-bat, 44 BdCL), comprising 315 unique bat–virus trials. We categorized host taxonomy, viral family (ICTV), and biogeographical congruence, to quantify temporal trends, taxonomic coverage, and host–virus pairing patterns. Results show stark underrepresentation: only 45 of ~1,500 bat species (3.2%) and 7 of 21 bat families appear in EIS. Pteropodidae, Vespertilionidae, Phyllostomidae, and Molossidae dominate studies, with *Rousettus aegyptiacus*, *Tadarida brasiliensis*, and *Eptesicus fuscus* most frequently trialed. Viral focus is uneven—Rhabdoviridae and Orthomyxoviridae (notably rabies [RABV] and influenza A [IAV]) account for many trials, and filoviruses are overrepresented in *Rousettus* sp. studies. By 2024, over half (57.8%) of trials used BdCL, and the proportion of live-bat experiments has declined in recent decades. Geographic coverage was uneven: high representation occurred in Arctic–Siberian, Eurasian, and North American regions, whereas Amazonian, Indo-Malayan, Australasian, Papua–Melanesian, and Madagascan regions were poorly represented. These biases constrain inference about host competence, immune diversity, and coevolutionary dynamics and risk overgeneralizing results across Chiroptera. These skews create a “mix-and-match” problem in which experimental pairings often do not reflect co-evolved host–virus relationships, limiting inference about host competence, immune tolerance, and zoonotic risk. To improve ecological relevance and generalizability, we recommend expanding taxonomic and geographic sampling, situating more work within virus native ranges, and integrating complementary *in vitro* and *in vivo* approaches alongside investments in regional biosafety infrastructure and standardized protocols. Addressing these critical gaps would better resolve the ecological and evolutionary determinants of susceptibility and tolerance in bats, improving mechanistic inference relevant to zoonotic emergence and conservation.

## 1 Introduction

Bats are frequently implicated as natural reservoir hosts for pathogens, including viruses that cause significant pathology in humans after spillover (1–3). Experimental infection studies (EIS) are a critical component of studying host status and specific bat-virus interactions (4–7). The outcomes of EIS may include information about: (1) infectivity and cell/organ tropism, (2), molecular mechanisms of viral entry and replication, (3) virulence of the virus, (4) the hosts’ immunological response to infection; (5) the presence and nature of viral shedding; and (6) whether the virus can naturally spread between individuals of the study species. Collectively, these findings can inform hypotheses about the nature of a particular bat-virus interaction and the potential reservoir host status of the study species.

Experimental infection studies are typically performed either in cell lines or in whole organisms and each type of experiment informs different aspects of the bat-pathogen interaction (8). Cell line or *in vitro* experiments provide controlled, high-throughput, mechanistic insight into molecular and cellular processes (e.g., receptor usage, replication kinetics, antiviral responses, genetic perturbations) that are often impossible to dissect or control *in vivo* (9–11). These results help inform and generate hypotheses and, further down the road, identify candidate pathways or targets that might be important for drug design. Whole-organism studies provide insight on host-virus interactions in the context of immune complexity (12), energy balance and thermoregulation (13) and tissue tropism (14) that shape real-world outcomes on the hosts’ responses. Using cell-line and whole-organism EIS in a complementary, synergistic workflow leverages the strengths of each system while mitigating their weaknesses (15). Combining approaches improves model selection and parameterization for computational models, strengthens reproducibility, and informs risk assessment and translational decisions [e.g., vaccine or therapeutic development (16, 17), spillover risk mitigation (18–20)]. Practically, this synergy reduces unnecessary animal use by focusing *in vivo* work on the most promising leads, optimizes resource allocation, and yields a more complete, biologically grounded picture of host–pathogen interactions than either approach alone (15, 21).

An important outcome of many bat-virus EIS is improved understanding of whether a given bat species responds to infection with a given virus in a manner that indicates susceptibility, immune resistance, or immune tolerance. These variable responses are presumably driven by the evolutionary relationship (or lack thereof) between a given species and a given pathogen (22–25). Bats that are susceptible to infection and that experience significant morbidity or even mortality in response to a particular virus are likely novel hosts for that pathogen. Bats that are resistant to viral infection are able to limit pathogen replication, a response that may suggest a relatively recent evolutionary relationship with the pathogen (26, 27). Finally, bats that are able to mitigate the health impacts caused by a pathogen burden display immunological tolerance, which suggests a long co-evolutionary relationship with the pathogen (28–30). For non-susceptible bat hosts, increasing data demonstrate that pathogens are controlled through a combination of tolerance and effective control over replication processes (31).

Because of the presumed co-evolutionary linkages between pathogens and the bat immune response, it is critical to consider biodiversity in both bat hosts and pathogens. With more than 1500 species spread across 21 families, bats exhibit greater morphological, anatomical, physiological, ecological, and phylogeographical diversity than any other mammalian order (32, 33). Diversity among the pathogens that are known to infect bats is even greater (34–36). Among the viruses, our understanding of phylogenetic and biological diversity is rapidly expanding, but much remains to be learned about this ubiquitous group (37, 38) members of which vary tremendously in size, shape, genomic material, replication cycle, and provenance (39). Understanding the impact of bat and viral diversity on the bat immunological response to infection is crucial (40, 41) and from this understanding it follows that not all bat-pathogen pairings are equally informative. To gain a better understanding of the current status of bat-virus EIS with regards to the nature of bat and virus species combinations, we conducted a systematic literature search for viral EIS in bats published between January 1900 and January 31st 2025, categorizing the studies into experimental infections in animal subjects and in bat-derived cell lines (BdCL). This study catalogs and quantifies the bat species (both for live bats and BdCL) used in viral EIS, evaluates the biogeographic congruence between experimental hosts and the viruses’ native ranges, and describes EIS efforts thus far in relation to the Chiroptera phylogenetic tree.

## 2 Methods

### 2.1 Data collection

We collated data from peer-reviewed primary research articles published from January 1900 to January 31st 2025 that included studies of live bats and/or BdCL that were experimentally infected with a virus. Our search string was “bat OR bats OR chiroptera (All Fields) AND vir* (All Fields) AND experimental* AND (infection OR inoculation) (All Fields)”, performed in Web of Science (all databases; Clarivate Analytics). To ensure complete coverage of BdCL studies, we complemented our results by including results from “bat OR bats OR chiroptera (All Fields) AND vir* (All Fields) AND infection OR inoculation (All Fields)” AND “cell line” OR “cell culture” OR “primary cells” OR “immortalization” (All Fields)”. This search mechanism, by definition, produced data for analysis from published studies. It did not therefore include an any negative unpublished findings nor did we attempt to assess pseudo replication or study quality.

### 2.2 Data curation

We used the AI tool Rayyan (42) and manually classified the papers as pertinent or non-pertinent to our study. We included any paper that made reference to bats and experimental viral infections and excluded papers that dealt with pathogen discovery unless they had also performed any experimental infections either with bats or BdCL. We also excluded from our analysis any Vesicular Stomatitis Virus (VSV) experimental infection in which VSV was used only as a vector for a transfection with proteins or genes from a different virus (**Supplementary Material**: PRISMA diagram).

### 2.3 Taxonomical check

After dividing the papers into the “live Bat” or “BdCL” categories (or occasionally, both), we inspected all papers and extracted the names of the bat species and virus used in each of the studies. Because our understanding of bat taxonomy is ever evolving, with frequent taxonomic revisions that can result in scientific names in older papers being incorrect, we checked the names of all bat species to ensure alignment with current taxonomic understanding using the Bat-Taxonomic-Alignment tool version 5 (January 2026) (43). All bat species were then categorized into their corresponding family following batnames.org (33). For the virus, we used the ICTV Virus database (https://ictv.global/) to categorize all viruses into their respective viral family. If a paper included more than one bat-virus pairing, we included all specific crossings as a unique bat-virus trial for any downstream analysis.

### 2.4 Geographical assessment

The extracted information was used to assess whether the bat species (or donor species in the case of BdCL) and the virus represented *biogeographically-congruent* experimental trials. We defined a *biogeographically-congruent* trial as one in which an experimental infection (either *in vivo* or *in vitro*) was conducted between entities that naturally occur in the same biogeographical region, using the 20 global regions and 11 realms as defined by Holt et al. (44). For example, if an *Artibeus jamaicensis* was experimentally infected with a Dengue virus (regardless of the serotype) this was considered a *biogeographically-congruent* trial crossing since this animal naturally occurs in areas where Dengue virus is naturally circulating. But if this same species were to be challenged with an *Orthoebolavirus*, this would be considered as *non*-*biogeographically-congruent* trial crossing because this virus does not circulate within the range of this species. All included papers and the decision database can be found in this GitHub repository (https://github.com/luisvqz/mix_and_match).

### 2.5 Map generation

We obtained the available distribution ranges in shapefile format for all bat species from the IUCN Red List of Threatened Species (IUCN 2026). We loaded these shapefiles (N = 1300) and estimated virtual bat assemblages and corresponding species richness by overlapping the species’ ranges with the zoogeographic regions defined by Holt et al. (44). We then calculated the percentage of species within each ecoregion that had been evaluated in any EIS. For species whose distributions spanned multiple ecoregion boundaries, the species was accounted for in all overlapping ecoregions (e.g., *Rousettus aegyptiacus*, *Tadarida brasiliensis*, and *Desmodus rotundus*). All spatial analyses were performed using ArcGIS Pro.

## 3 Results

Our search yielded a total of 545 papers after removing duplicates. After manual filtering, our consolidated database included 82 papers for bats and 45 papers for BdCL, and 2 for both, collectively generating 315 different bat-virus pairings (**Supplementary Table 1**). Our findings reveal that 132 (41.9%) EIS were performed on live bats while 183 (58.1%) were performed on BdCL. Three studies that used organoids were classified as BdCL studies, noting that the use of organoids will likely increase and hold the promise of adding additional levels of information. We found that 45 species of bats have been trialed in viral EIS, BdCL trials (n=34) have been performed in more bat species than in live bats (n=28), with an extremely sharp increase in the number of studies since the first study in 2003 (**Figure 1**).

**Figure 1:**
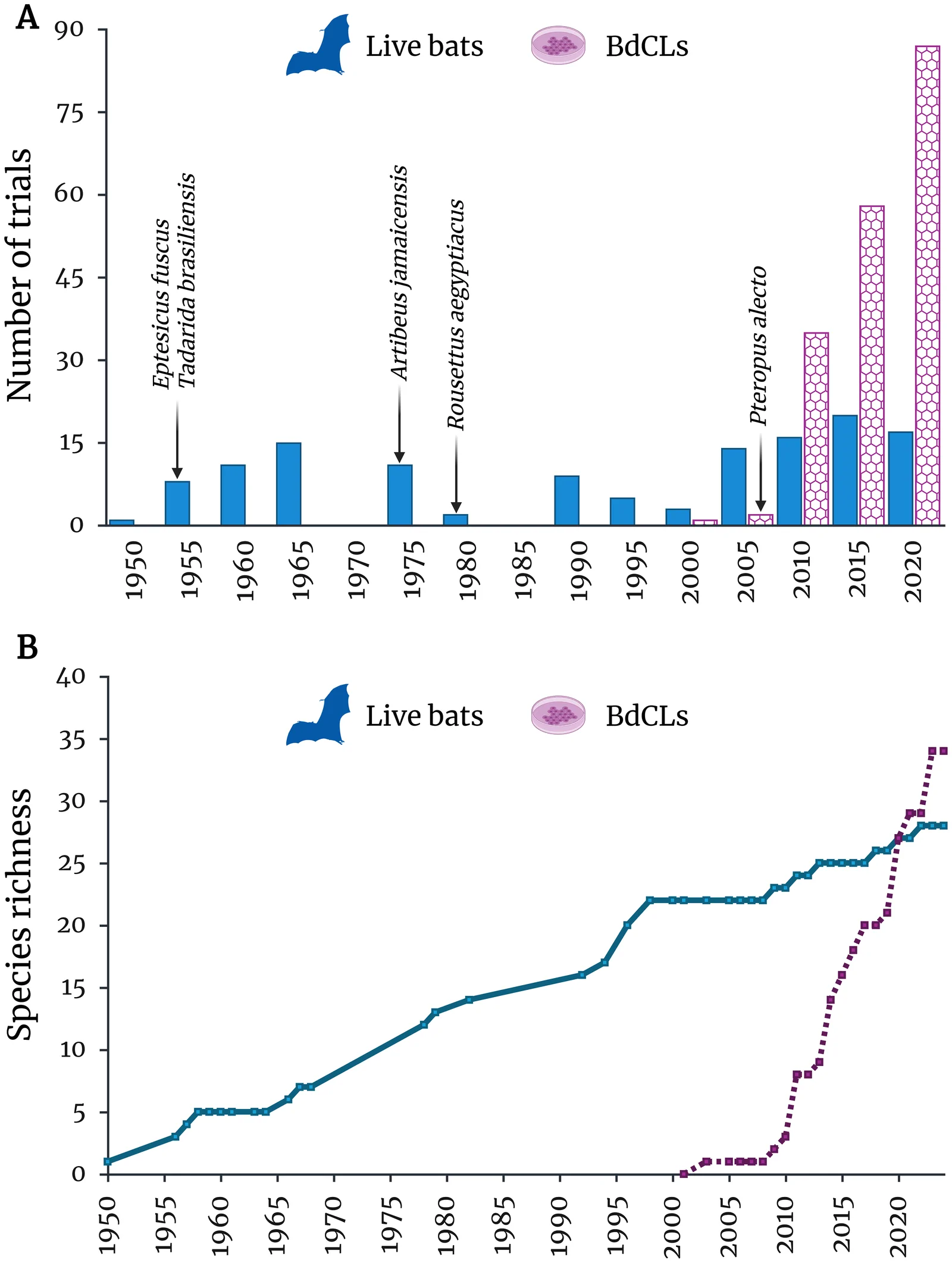
Experimental infection studies (EIS) trials performed in live bats and in BdCL between 1950 and 2025 **(A)**; arrows indicate the first EIS trial for each of six focal species (*Eptesicus fuscus*, *Tadarida brasiliensis*, *Artibeus jamaicensis*, *Rousettus aegyptiacus*, *Pteropus alecto* and *Eidolon helvum*). **(B)** Species accumulation curves for species included in these EIS categories plotted against year of publication. Created in BioRender.

We found EIS that included species only from 7 of the 21 bat families (33%): Pteropodidae (n=132, 41.9%), Vespertilionidae (n=70, 22.4%), Phyllostomidae (n=49, 15.5%), Molossidae (n=41, 13.1%), Miniopteridae (n=12, 3.8%), Rhinolophidae (n=10, 3.2%) and Hipposideridae (n=1, 0.3%) (**Figures 2A, B**). Although these 7 families are the most speciose (with the exception of Emballonuridae), collectively accounting for 1365/1500 or 91.0% of all bat species, EIS studies have not been conducted in bats in the additional 14 bat families. Species coming from these 7 families have been evaluated against 60 different viruses, including Rhabdoviridae (n=14), Filoviridae (n=9), Flaviviridae (n=8), Paramyxoviridae (n=7), Retroviridae (n=4), Coronaviridae (n=3), and Orthomyxoviridae, Togaviridae, Picornaviridae, Sedoreoviridae, Phenuiviridae, Arenaviridae, Orthoherpesviridae, Adenoviridae, Bunyaviridae, Peribunyaviridae, Reoviridae with two or less viral species evaluated. This bias is even more evident when we look only at EIS with live bats, where only four families (Vespertilionidae, Pteropodidae, Phyllostomidae and Molossidae) have been included (**Figure 2C**).

**Figure 2:**
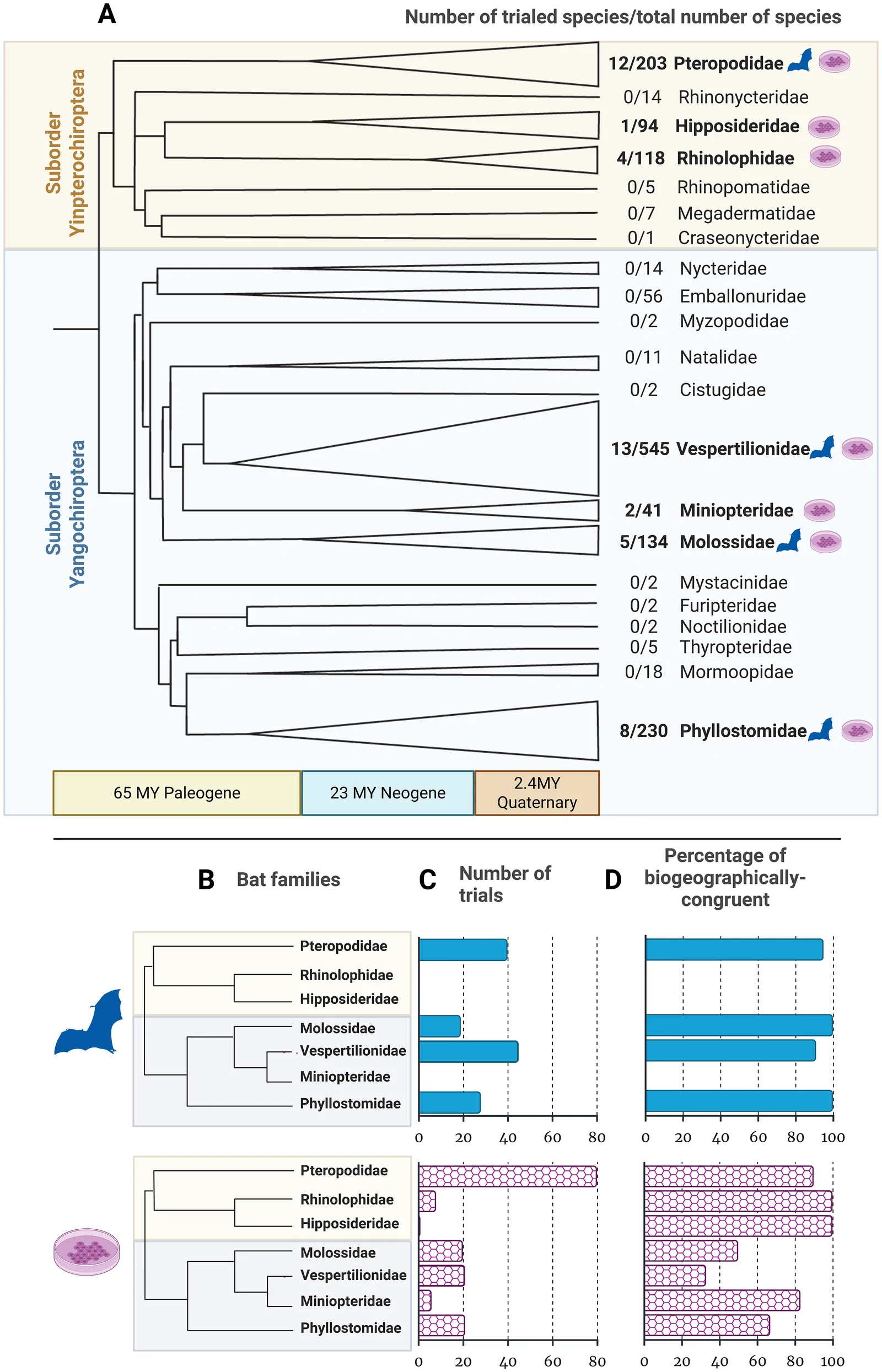
**(A)** Illustrative rendering of phylogenetic relationships among bat families based on Upham et al. (45) with modifications from (46) for estimating some relative dates and the placement of the family Rhinonycteridae, data from which were missing from the original paper, the placement of the family Myzopodidae remains uncertain and deeper relationships within the tree are not fully resolved. Relative age of family level diversification is shown for families with greater than 10 described species, and width of the tip of each family-level branch is relative to the number of described species. The number of bat species for which any experimental infection study (EIS) was conducted are indicated, followed by the number of described species currently recognized in each family (33); icons indicate either live animal or bat-derived cell lines (BdCL), **(B)** Truncated phylogenetic tree for the 7 families of bats in which EIS have been performed, **(C)** Number of unique bat species-virus EIS per family for live bats (top) and BdCL (bottom). **(D)** Percentage of trials within each bat family that were categorized as biogeographically-congruent EIS (either *in vivo* or *in vitro*), that is, those conducted between entities that naturally occur in the same biogeographical region, using the 20 global regions and 11 realms as defined by Holt et al. (44). Created in BioRender.

*Rousettus aegyptiacus* (n=47, 15%) was the most trialed species, followed by *Tadarida brasiliensis* (n=35, 11.2%), *Eptesicus fuscus* (n=30, 9.6%), *Artibeus jamaicensi*s (n=20, 6.4%), and *Pteropus alecto* (n=15, 4.8%) (**Supplementary Table 2**). From the viral perspective, the most trialed viruses are RABV (n=26, 8.3%) and IAV (n=26, 8.3%) trialed against 8 and 12 bat species respectively (**Figure 2C**). The most trialed pair have been *Rousettus aegyptiacus*-MARV *(Orthomarburgvirus marburgense;* n=14) followed by *Tadarida brasiliensis*-RABV (*Lyssavirus rabies*; n=7), *Eptesicus fuscus*-RABV (n=6), *Rousettus aegyptiacus*-EBOV (*Orthoebolavirus zairense*; n=6), *Carollia perspicillata*-IAV (*Alphainfluenzavirus influenzae*; n=5), *Desmodus rotundus*-RABV (n=5) and *Pteropus alecto*-HeV (*Henipavirus hendraense;* n=5) (**Figure 3A–C**).

**Figure 3:**
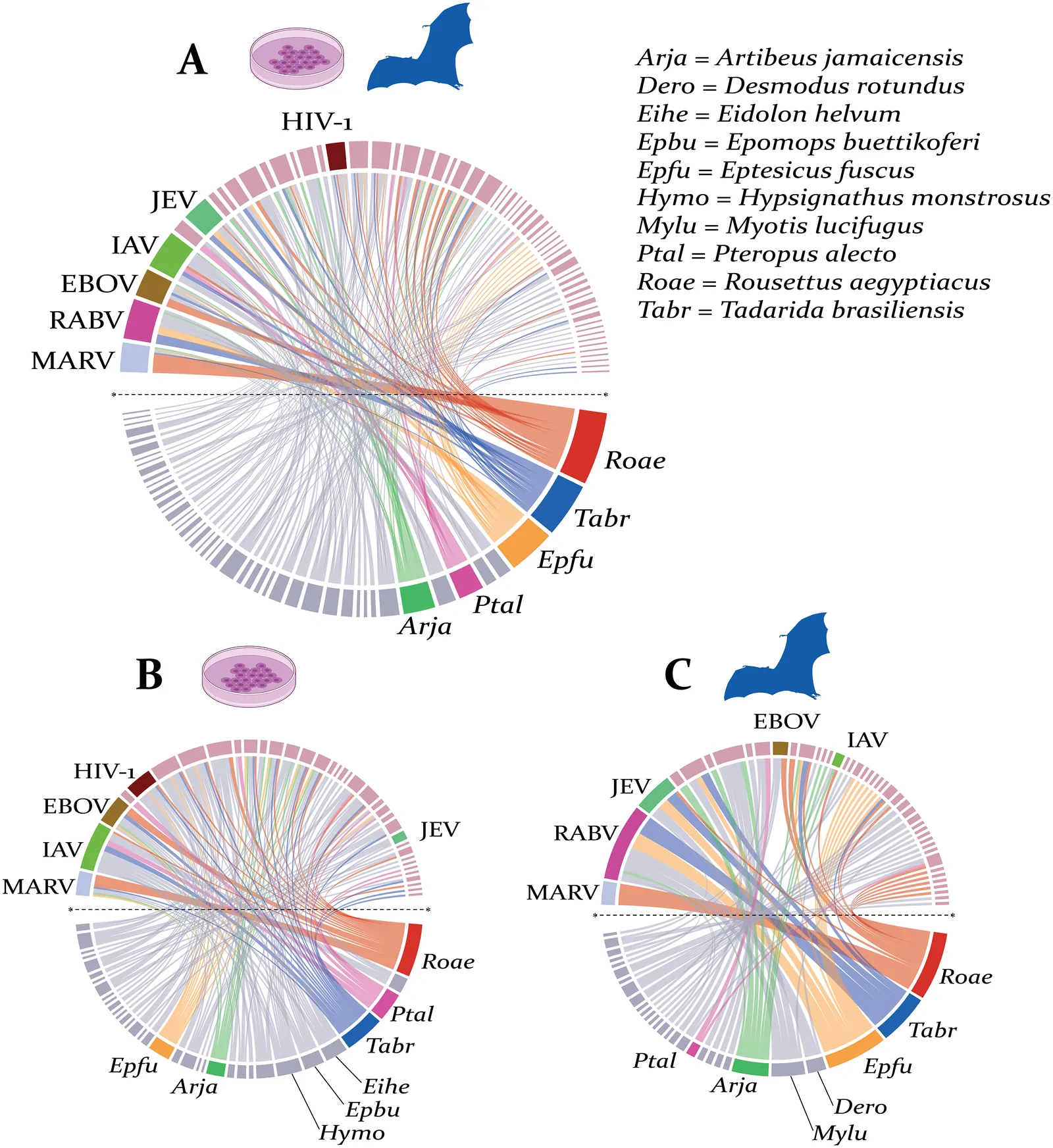
Connections between the six most trialed species and the six most trialed viruses for both live bats and BdCL **(A)**, BdCL only **(B)**, and live bats only **(C)**. Each chord represents a separate trial conducted between that species and the virus.

In terms of geographical representation, we found that there is a heavier bias of *biogeographically-congruent* trials for BdCL 96 ± 5% (n=155) in comparison to live bats, 75 ± 26% (n=132) (**Figure 2D**). Although not evenly distributed across all families, we do see a heavy bias of BdCL trials performed with bats of the Pteropodidae family (42% of all BdCL trials). For this comparison, we excluded from the dataset the globally distributed human-exclusive pathogens (HIV-1 and HSV-1 (*Simplexvirus humanalpha1*)) and one rodent-exclusive pathogen (MuLV; *Gammaretrovirus murleu*).

We found that Arctico-Siberian (19%, n=6), Eurasian (13%, n-14), Japanese (13%, n=3), Saharo-Arabian (12%, n=12), and North American (11%, n=5) were the regions with highest percentages of the accounted species had been included in any EIS trialed. In contrast, Amazonian (3%, n=8), Australian (3%, n=2), Indo-Malayan (3%, n=6), South American (3%, n=5), Papua-Melanesian (1%, n=2) and Madagascan (0%, n=0) are the ecoregions with lower percentage of coverage according to our geographical analysis.

## 4 Discussion

The diverse diets, physiology, and habitats of bats expose them to different pathogens, driving the evolution of specialized immune systems that can effectively respond to these threats (30, 47–49). This has resulted in remarkable immune resilience, allowing bats to coexist with and exhibit signals of immunological resistance and/or tolerance to numerous (but not all) viruses (29, 31, 50). Additionally, the varied climates and geographic features across the globe have shaped distinct physiological adaptations, such as differences in flight morphology, thermoregulatory mechanisms, and immune responses, allowing different bat species to efficiently exploit their particular niches (32, 49). This rich tapestry of adaptability highlights the importance of considering the unique natural history of each bat species, influenced by their habitats, evolutionary histories, and immune system developments.

Many studies have highlighted the value of live bat populations in research, particularly in understanding the ecological and biological factors influencing bat health (51–54). However, the use of BdCL offers a unique advantage, as it allows for the implementation of multiple comparisons across various species and conditions (26, 55, 56).

### 4.1 BdCL trials have surpassed the number of trials with live bats

Before the 1990s, research predominantly centered on a handful of New World species (*Myotis lucifugus*, *Eptesicus fuscus, Tadarida brasiliensis* and *Desmodus rotundus*). As awareness of the importance of biodiversity in disease dynamics has grown, there has been a marked shift towards a more inclusive experimental design that moves beyond just a few model bat species (15, 57). This change allows for a broader incorporation of aspects of bat ecology and biodiversity into study design and the interpretation of results. This greater body of studies has in large part been facilitated by the use of BdCL as a proxy for the donor species in experimental infection studies. Our results highlight how the proportion of studies that make use of BdCL has increased over the last 25 years (**Figure 1B**), with the majority of EIS now utilizing BdCL.

Several practical and regulatory constraints explain why live bat studies disproportionately involve fewer species. Many bat taxa are difficult to capture, transport, acclimate, and maintain in captivity due to dietary, roosting, and social requirements. Securing permits for capture, transport (especially internationally), and experimental work can be lengthy and restrictive, especially for protected or endemic species. High-containment requirements for many zoonotic viruses (BSL-3/4), specialized infrastructure, and time-consuming quarantine and welfare considerations further raise logistical, financial, and ethical concerns (58).

### 4.2 Bat diversity is poorly represented across EIS

Experimental infection studies (EIS) of bat–virus interactions exhibit pronounced taxonomic bias: only 4 of 21 family-level clades have been represented in live bats-virus experiments and only 7 of 21 bat families have been included in BdCL studies (**Figure 2**). Across these 7 families, a total of 45 species have been studied, less than 5.0% of species in each of these families. The exception is found in the Old World fruit bats (Pteropodidae), in which 5.9% of species have been included - not surprising given their role as hosts of Marburg virus and the Henipah viral species [**Figure 2A**; (5, 6)]. We were surprised at the limited number of Rhinolophid species (4/118; 3.4%) studied given the diversity of SARS-related coronaviruses in this lineage. This relatively narrow taxonomic sampling, both across and within families, limits inference about host competence, immunological diversity, and the evolutionary determinants of susceptibility across Chiroptera (**Figure 4**). Unfortunately, results from the 7 studied lineages are often extrapolated to broad conclusions about “bats” as a whole, despite substantial physiological, ecological, and phylogenetic heterogeneity among families and genera (32, 59). Our demonstrated biases and data emerging from comparative genome analyses and the body of literature exploring bat immunity make it clear that bats are not a monolithic group with regard to host-pathogen interactions (25, 54).

**Figure 4:**
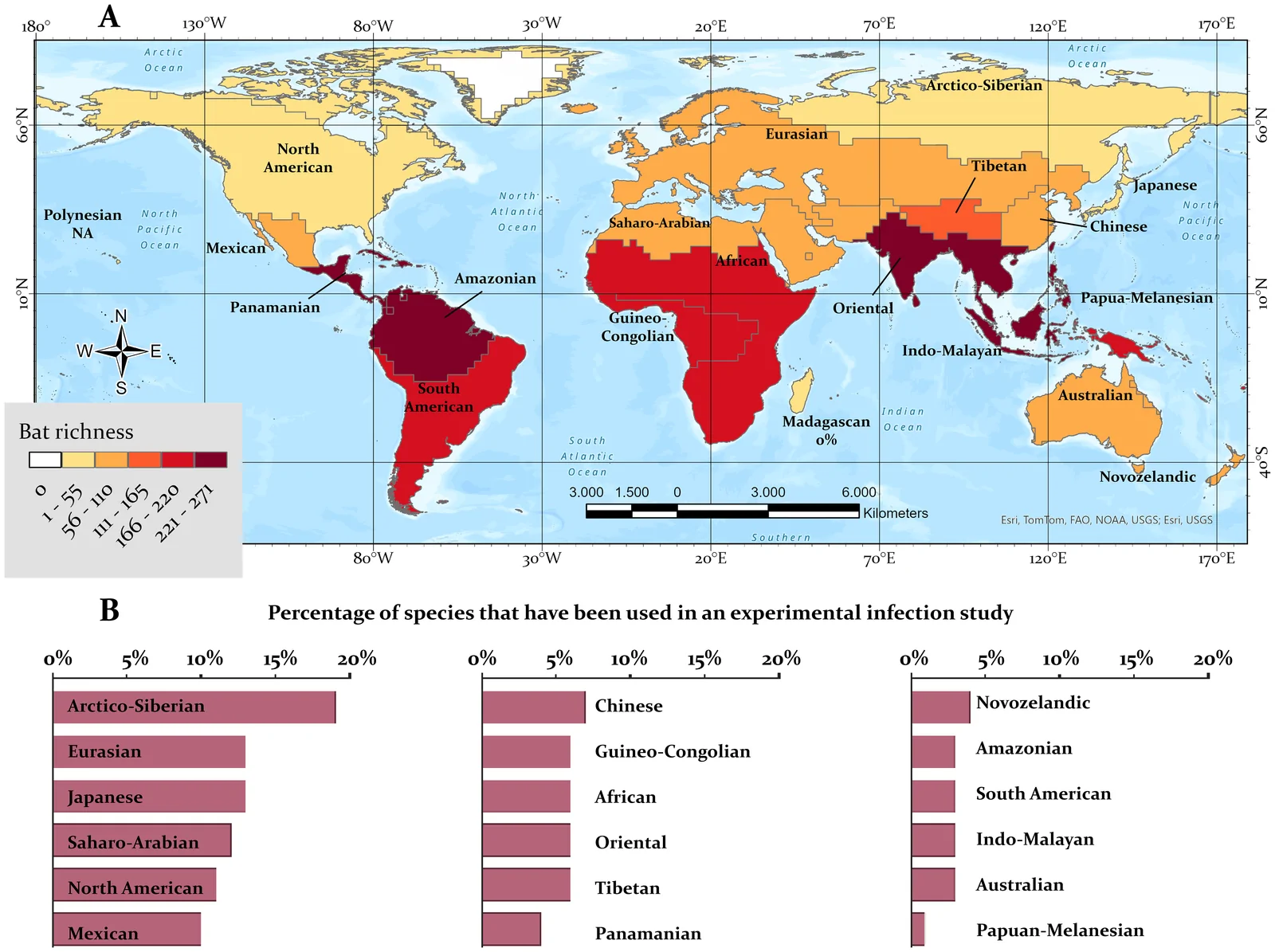
**(A)** Bat species richness across all Holt et al. (44) ecoregions. Data were obtained from the range polygons from International Union for the Conservation of Nature (consulted in March 2026). The IUCN database included 1300 species (86.7% of extant species) but newly described or data deficient species are not yet included in the database. The percentages represent the proportion of species present in that region that have been utilized in any EIS (either live bats or BdCL). **(B)** Percentage of species that have been used in an experimental infection study (EIS) for each ecoregion; it is worth noting that the best performance was in the Artico-Siberian ecoregion (19%) while some of the species rich ecoregions (i.e. Amazonian, Panamanian and Indo-Malayan) have low (<5%) species representation.

Beyond the broad-brush phylogenetic biases described above, the logistical challenges of both live bat and BdCL EIS have resulted in a “backyard” sampling bias that further constrains the ecological relevance of findings. Researchers frequently use locally available species—commonly from North America—to test viruses originating from other biogeographic regions. This practice produces geographic and evolutionary mismatches between experimental hosts and the natural reservoir or spillover host of interest, meaning measured responses often reflect novel hosts rather than species co-evolved with the virus *in situ*. Such mismatches can mischaracterize transmission potential, virulence, and immune dynamics that would occur in the virus’s native host community, in which signals of resistance and/or tolerance could be expected. It is important to note, however, that intentional phylogeographic mismatches between bat and viral species can produce important knowledge about responses of novel bat hosts and answer timely questions, such as which bat species might be susceptible to or competent hosts of SARS-CoV-2 (60–71).

To overcome the barriers to broader taxonomic testing and to reduce ecological and evolutionary mismatches between experimental hosts and viruses, investment is needed for in-country biosafety infrastructure, high-containment facilities (when potential human pathogens are being studied), quarantine capacity, and standardized protocols located within biodiversity hotspots (**Figure 4**). Building such infrastructure—often in regions of the Global South where bat diversity and viral reservoir richness are highest—would accelerate research (72). Additionally, the establishment and further development of these international cooperations will boost the ecological relevance and generalizability of results, while simultaneously building up and supporting local capacity, equitable scientific collaboration, and regional biosafety readiness (73).

Addressing these gaps requires deliberate expansion of taxonomic breadth and methodological standardization: prioritizing inclusion of underrepresented families and geographically diverse populations, integrating host phylogeny and ecology into experimental design, and reporting host and strain-level virus metadata (3, 20, 74). Coordinated networks and shared repositories for biospecimens, viral isolates, and protocols would reduce redundancy and allow meta-analytic synthesis, while ethical and biosafety safeguards must guide sampling to minimize harm and risk. Only by pairing broader, phylogenetically informed sampling with transparent data practices can EIS deliver robust, generalizable insights into bat–virus ecology and zoonotic risk.

### 4.3 BdCL and live bat studies can be synergistically informative

The development of bat-derived cell lines has proven to be a pivotal advancement in bat research methodologies as seen by the exponential growth of studies since the first BdCL EIS paper in 2003 (**Figure 1A**). These cell lines provide accessible and consistent platforms for experimental assays, promoting the comparability of results across different studies (57). However, the ongoing refinement of BdCL must take into account that these cell lines, lacking integrative immunological responses, may not accurately reflect all of the complex interactions seen in live animals (75). BdCL behave differently than live bats in similar situations due to their lack of complete immunological cascades. Even organoids, the most advanced developments in cell-lines (76, 77), cannot fully replicate complex biological systems including relevant immune-epithelial interactions (78, 79). EIS performed on BdCL recreate only the infection dynamics of a particular tissue under isolated and controlled conditions (75), but more importantly, not all tissues can be successfully represented with BdCL. In the real world, animals do not live in a vacuum as cell cultures do; and there are endless feedback loops that modulate, enhance or even nullify each other in the immunological cascades (80). These modified conditions can lead to different or even misleading responses to pathogens and stressors compared to what would happen in “natural” infections scenarios (81–83).

A comprehensive understanding of bat immunity and host-pathogen interactions requires a combination of *in vitro* and *in vivo* studies. Isolated cell cultures might provide insight into specific components of antiviral responses, but they do not fully replicate the integrated responses of the entire organism (31, 54). This is especially true for bats. As the only flying mammals, both their physiology and their ecology are unique, and we have much still to learn. Some aspects of their physiology and how they may affect immune processes and reservoir host status remain to be understood (84, 85). Chief among these is the profound daily and/or seasonal heterothermy displayed by most species, even in the tropics (86–88). Incorporating ecological factors, such as habitat and microclimate preference, co-roosting with sympatric species, and migration is equally vital when studying bat immune mechanisms (40, 51). By conducting experiments in more natural settings, researchers can capture the full spectrum of immune behaviors influenced by ecological dynamics, physiology, and social interactions within bat populations. This holistic approach, in which the strengths of both *in vitro* and *in vivo* studies are maximized creates a positive feedback loop: as researchers continue to explore additional applications and adaptations of these technologies while staying aware of their limitations, they unlock further insights into bat biology and improve methodologies (54, 57). This iterative process significantly enhances the robustness of bat disease ecology research.

The best examples of what is possible come from “model” bat species, such as the well-studied *R. aegyptiacus.* This widely distributed Old World bat in the family Pteropodidae, has been the subject of various EIS conducted across multiple viral families (**Figure 1A**; **Supplementary Table 1**)- with more live bat and BdCL studies than any other species (**Figures 1B–D**). These studies have included viruses from the Filoviridae (31), Flaviviridae (3), Orthomyxoviridae (2), Paramyxoviridae (3), Phenuiviridae (3), Retroviridae (3), Coronaviridae (1) and, Rhabdoviridae (1) families (**Supplementary Table 1**). The extensive research on Filoviridae in this cavernicolous bat was initially driven by early epidemiological connections between Marburg virus and caves containing this species. That *R. aegyptiacus* is a natural reservoir host for Marburg is now well established (24, 89, 90) and strong evidence exists for it also hosting Sosuga Virus and Kasokero Virus (6, 91–93). The development of tools and molecular resources for this study species, including BdCL (94), an annotated genome (95), and annotated transcriptomes (96), including at the single cell level for leucocytes (97), have greatly facilitated our understanding of host-pathogen interactions in this important viral reservoir. Additionally, the development of research infrastructure within the large natural range of this species (e.g., the Uganda Virus Research Institute (UVRI); Lokey Preclinical Research Facility (MAMTEK) in Israel) has facilitated ecological and immunological understanding. Finally, the existence of captive and biosecure research colonies, such as the one at the US Centers for Disease Control and Prevention, have greatly facilitated EIS, allowing for controlled, safe, and comprehensive research (e.g., (7, 24, 90, 98).

With recent advances in techniques and tools, our understanding of bat-virus interactions is increasing rapidly. However, our identified “mix-and-match” problem—in which bats are often treated as a monolithic group while combining experimental viruses and hosts without regard to their co-evolutionary history—undermines mechanistic inference. Comparative and predictive studies therefore risk conflating lineage-specific traits with general bat-level patterns, reducing the utility of EIS for understanding zoonotic emergence across diverse bat clades. By actively working to expand the number and phylogeographic diversity of bat species included, by thoughtful consideration of which bat-virus combinations are most important to study, and by including both *in vitro* and *in vivo* experiments, our ability to understand the bat immune system and the complexity of bat-host interactions will be optimized. The future is ripe: never has bat immunology been more relevant in both scientific and public fora. With new cell-derived systems (e.g., organoids) and expanding international collaboration networks (e.g., GBatNet, Bat1K), we stand at a turning point—now we must ensure growth is not just rapid but also representative of bats’ phylogenetic and geographic diversity.
