# Supplementary material for: Mismatched and underrepresented: bat–virus diversity and phylogeographic bias in experimental infection studies
Source: Front Immunol. 2026 Sep 14;17:1901431. doi: 10.3389/fimmu.2026.1901431 (PMC13617609; doi:10.3389/fimmu.2026.1901431)
Supplement: Supplementary file 1 [file Table1.docx]

**PRISMA Diagram**

Haddaway, N. R., Page, M. J., Pritchard, C. C., & McGuinness, L. A. (2022). PRISMA2020: An R package and Shiny app for producing PRISMA 2020-compliant flow diagrams, with interactivity for optimised digital transparency and Open Synthesis Campbell Systematic Reviews, 18, e1230. <https://doi.org/10.1002/cl2.1230>

**Supplemental Table 1:**

| **Bat family** | **Bat species** | **Live Bats trials** | **BdCL trials** | **Viral families** | **References** |
| --- | --- | --- | --- | --- | --- |
| Pteropodidae | *Eidolon helvum* | 2 | 10 | Flaviviridae, Orthomyxoviridae, Paramyxoviridae, Phenuiviridae | 84, 71, 59, 60, 116, 97, 112, 63, 47 |
|  | *Eonycteris spelaea* | 1 | 6 | Coronaviridae, Orthoherpesviridae, Orthomyxoviridae, Sedoreoviridae | 124, 115, 93 |
|  | *Epomophorus crypturus* |  | 3 | Retroviridae | 116 |
|  | *Epomophorus wahlbergi* | 1 |  | Filoviridae | 25 |
|  | *Epomops buettikoferi* |  | 9 | Filoviridae, Flaviviridae, Orthomyxoviridae | 71, 73, 63 |
|  | *Hypsignathus monstrosus* |  | 12 | Filoviridae, Flaviviridae, Orthomyxoviridae, Paramyxoviridae | 95, 71, 59, 60, 73, 63 |
|  | *Pteropus alecto* | 3 | 13 | Coronaviridae, Flaviviridae, Orthoherpesviridae, Orthomyxoviridae | 48, 102, 76, 94, 61, 122, 93, 41 |
|  | *Pteropus dasymallus* |  | 5 | Flaviviridae, Picornaviridae, Retroviridae | 101, 116 |
|  | *Pteropus poliocephalus* | 3 |  | Paramyxoviridae | 26, 36, 28 |
|  | *Pteropus vampyrus* | 3 | 3 | Paramyxoviridae, Togaviridae | 48, 107, 67, 11 |
|  | *Rousettus aegyptiacus* | 24 | 24 | Coronaviridae, Filoviridae, Flaviviridae, Orthomyxoviridae | 113, 95, 110, 62, 96, 29, 103, 104, 50, 99, 68, 73, 116, 79, 128, 70, 44, 21, 69, 86, 74, 63, 85, 77, 82, 72, 89, 47 |
|  | *Rousettus leschenaultii* | 3 | 7 | Coronaviridae, Flaviviridae, Picornaviridae, Retroviridae | 46, 101, 116, 20, 106, 45 |
| Hipposideridae | *Hipposideros caffer/ruber* |  | 1 | Phenuiviridae | 47 |
| Rhinolophidae | *Rhinolophus ferrumequinum* |  | 5 | Flaviviridae, Picornaviridae, Retroviridae | 101, 116 |
|  | *Rhinolophus landeri* |  | 3 | Orthomyxoviridae, Phenuiviridae | 62, 71, 47 |
|  | *Rhinolophus lepidus* |  | 1 | Coronaviridae | 119 |
|  | *Rhinolophus sinicus* |  | 1 | Paramyxoviridae | 66 |
| Vespertilionodae | *Antrozous pallidus* | 1 |  | Rhabdoviridae | 4 |
|  | *Eptesicus fuscus* | 20 | 10 | Coronaviridae, Filoviridae, Flaviviridae, Picornaviridae | 9, 55, 2, 100, 39, 13, 75, 12, 73, 116, 35, 37, 7, 40, 56, 54, 17, 109, 32 |
|  | *Eptesicus nilssonii* |  | 3 | Retroviridae | 116 |
|  | *Eptesicus serotinus* | 1 |  | Rhabdoviridae | 43 |
|  | *Myotis brandtii* | 3 |  | Rhabdoviridae | 23 |
|  | *Myotis daubentonii* | 4 | 4 | Flaviviridae, Orthomyxoviridae, Phenuiviridae, Rhabdoviridae | 62, 23, 63, 38, 47 |
|  | *Myotis davidii* |  | 2 | Adenoviridae, Paramyxoviridae | 52, 66 |
|  | *Myotis lucifugus* | 11 |  | Flaviviridae, Paramyxoviridae, Rhabdoviridae | 9, 2, 12, 1, 5, 58, 6, 16 |
|  | *Myotis myotis* | 1 | 1 | Bunyaviridae, Rhabdoviridae | 120, 15 |
|  | *Nyctalus noctula* |  | 1 | Filoviridae | 92 |
|  | *Perimyotis subflavus* | 1 | 2 | Flaviviridae, Orthomyxoviridae, Rhabdoviridae | 2, 63 |
|  | *Pipistrellus pipistrellus* | 3 | 1 | Rhabdoviridae, Phenuiviridae | 62, 24 |
|  | *Pipistrellus sp.* |  | 1 | Phenuiviridae | 47 |
| Miniopteridae | *Miniopterus fuliginosus* |  | 8 | Flaviviridae, Picornaviridae, Retroviridae | 101, 116 |
|  | *Miniopterus schreibersii* |  | 4 | Filoviridae, Retroviridae | 117 |
| Molossidae | *Mops plicatus* |  | 1 | Peribunyaviridae | 108 |
|  | *Molossus sinaloe* |  | 1 | Flaviviridae | 78 |
|  | *Mops condylurus* | 1 | 2 | Filoviridae | 92, 25, 88 |
|  | *Mops pumilus* | 1 |  | Filoviridae | 25 |
|  | *Tadarida brasiliensis* | 17 | 18 | Coronaviridae, Filoviridae, Flaviviridae, Orthomyxoviridae | 9, 31, 53, 71, 12, 114, 73, 5, 87, 116, 4, 6, 37, 10, 16, 3, 126, 14, 63, 8, 123, 32 |
| Phyllostomidae | *Artibeus intermedius* | 2 |  | Flaviviridae, Rhabdoviridae | 57, 81 |
|  | *Artibeus jamaicensis* | 12 | 9 | Arenaviridae, Coronaviridae, Filoviridae, Flaviviridae | 19, 111, 64, 18, 125, 127, 73, 121, 78, 91, 80, 51, 90, 30 |
|  | *Artibeus lituratus* | 2 |  | Togaviridae | 19, 18 |
|  | *Carollia perspicillata* | 1 | 8 | Coronaviridae, Orthomyxoviridae, Phenuiviridae | 118, 62, 98, 71, 105 |
|  | *Carollia subrufa* | 2 |  | Togaviridae | 19, 18 |
|  | *Desmodus rotundus* | 5 | 1 | Flaviviridae, Rhabdoviridae | 34, 78, 65, 27, 83, 33 |
|  | *Phyllostomus discolor* | 2 |  | Togaviridae | 19, 18 |
|  | *Sturnira parvidens* | 2 | 3 | Coronaviridae, Orthomyxoviridae, Togaviridae | 19, 18, 105 |

**Screened Papers**

1. Reagan, R., Smith, E. & Brueckner, A. Studies of Newcastle Disease Virus (NDV) Propagated in the Cave Bat (*Myotus lucifugus*). Proceedings of the Society for Experimental Biology and Medicine 75, 691–692 (1950). doi.org/10.3181/00379727-75-18307

2. Stamm, D., Kissling, R. & Eidson, M. Experimental Rabies Infection in Insectivorous Bats. Journal of Infectious Diseases 98, 10–14 (1956). doi.org/10.1093/infdis/98.1.10

3. Sulkin, S., Krutzsch, P., Wallis, C. & Allen, R. Role of Brown Fat in Pathogenesis of Rabies in Insectivorous Bats (*Tadarida-B-Mexicana*). Proceedings of the Society for Experimental Biology and Medicine 96, 461–464 (1957). doi.org/10.3181/00379727-96-23507

4. Burns, K., Shelton, D. & Grogan, E. Bat Rabies - Experimental Host Transmission Studies. Annals of the New York Academy of Sciences-Series 70, 452–466 (1958). doi.org/10.1111/j.1749-6632.1958.tb35403.x

5. Sulkin, S., Krutzsch, P., Allen, R. & Wallis, C. Studies On the Pathogenesis of Rabies in Insectivorous Bats .1. Role of Brown Adipose Tissue. Journal of Experimental Medicine 110, 369–388 (1959). doi.org/10.1084/jem.110.3.369

6. Sulkin, S., Allen, R., Sims, R., Krutzsch, P. & Kim, C. Studies On thePathogenesis of Rabies in Insectivorous Bats .2. Influence of Environmental Temperature. Journal of Experimental Medicine 112, 595–617 (1960). doi.org/10.1084/jem.112.4.595

7. Dempster, G., Spencer, W. & Grodums, E. Experimental Coxsackie B-3 Infection in Hibernating Squirrel and Bat. Canadian Journal of Microbiology 7, 587+ (1961). doi.org/10.1139/m61-068

8. Sims, R., Sulkin, S. & Allen, R. Studies On Pathogenesis of Rabies in Insectivorous Bats .3. Influence of Gravid State. Journal of Infectious Diseases 112, 17+ (1963). doi.org/10.1093/infdis/112.1.17

9. Sulkin, S., Allen, R. & Sims, R. Studies of Arthropod-Borne Virus Infections in Chiroptera .1. Susceptibility of Insectivorous Species to Experimental Infection with Japanese B and St Louis Encephalitis Viruses. The American Journal of Tropical Medicine and Hygiene 12, 800+ (1963). doi.org/10.4269/ajtmh.1963.12.800

10. Sulkin, S., Allen, R. & Sims, R. Studies of Arthropod-Borne Virus Infections in Chiroptera .2. Experiments with Japanese B + St Louis Encephalitis Viruses in Gravid Bat Evidence of Transplacental Transmission. The American Journal of Tropical Medicine and Hygiene 13, 475+ (1964). doi.org/10.4269/ajtmh.1964.13.475

11. Shah, K. & Daniel, R. Attempts At Experimental Infection of Indian Fruit-Bat Pteropus giganteus with Chikungunya and Dengue 2 Viruses and Antibody Survey of Bat Sera for Some Viruses. Indian Journal of Medical Research 54, 714+ (1966).

12. Sulkin, S., Allen, R. & Sims, R. Studies of Arthropod-Borne Virus Infections in Chiroptera .3. Influence of Environmental Temperature On Experimental Infection with Japanese B and St Louis Encephalitis Viruses. The American Journal of Tropical Medicine and Hygiene 15, 406+ (1966). doi.org/10.4269/ajtmh.1966.15.406

13. Sulkin, S. E., Allen, R., Sims, R. & Singh, K. V. Studies of Arthropod-Borne Virus Infections in Chiroptera. Iv. theImmune Response of the Big Brown Bat (*Eptesicus fuscus*) Maintained at Various Environmental Temperatures to Experimental Japanese B Encephalitis Virus Infection. The American Journal of Tropical Medicine and Hygiene (1966). doi.org/10.4269/ajtmh.1966.15.418

14. Baer, G. M. & Bales, G. L. Experimental Rabies Infection in theMexican Freetail Bat. J Infect Dis 117, 82–90 (1967). doi.org/10.1093/infdis/117.1.82

15. Kolman, J., Marhoul, Z. & Malkova, D. Experimental Infection of Bat *Myotis myotis* Borkhausen with Tahyna Virus. Journal of Hygiene Epidemiology Microbiology and Immunology 11, 125+ (1967). PMID: 6070328

16. Middlebrooks, B., Allen, R. & Sulkin, S. Effects of 6-Thioguanine and Endotoxin On Experimental St Louis and Japanese B Encephalitis Virus Infections in Chiroptera. Proceedings of the Society for Experimental Biology and Medicine 127, 886+ (1968). doi.org/10.3181/00379727-127-32828

17. Leonard, L. L., Allen, R. & Sulkin, S. E. Bat Immunoglobulins formed in Response to Experimental Japanese B Encephalitis (JBE) Virus Infection. The Journal of Immunology 101, 1168–1175 (1968). doi.org/10.4049/jimmunol.101.6.1168

18. Seymour, C., Dickerman, R. & Martin, M. Venezuelan Encephalitis-Virus Infection in Neotropical Bats .2. Experimental Infections. American Journal of Tropical Medicine and Hygiene 27, 297–306 (1978). doi.org/10.4269/ajtmh.1978.27.297

19. Seymour, C. & Dickerman, R. Venezuelan Encephalitis-Virus Infection in Neotropical Bats .3. Experimental Studies On Virus Excretion and Non-Arthropod Transmission. American Journal of Tropical Medicine and Hygiene 27, 307–312 (1978). doi.org/10.4269/ajtmh.1978.27.307

20. Banerjee, K., Ilkal, M., Bhat, H. & Sreenivasan, M. Experimental Viremia with Japanese Encephalitis-Virus in Certain Domestic and Peridomestic Vertebrates. Indian Journal of Medical Research 70, 364–368 (1979). PMID: 231573

21. Addy, P., Lule, M. & Sekyalo, E. Experimental-Infection of *Roussettus aegyptiacus* E, Geoffroy (Chiroptera) with Japanese-B-Encephalitis Virus. Annales De Virologie 133, 133–137 (1982). doi.org/10.1016/S0769-2617(82)80030-0

22. Lord, R., Dechaurell, A. & Campos, E. Experimental-Infection of Vampire Bats with Foot-and-Mouth-Disease Virus. Journal of Wildlife Diseases 22, 413–414 (1986). doi.org/10.7589/0090-3558-22.3.413

23. Botvinkin, A., Kuzmin, I. & Chernov, S. Experimental-Infection of Bats with Lyssaviruses Serotype-1 and Serotype-4. Voprosy Virusologii 37, 215–218 (1992). PMID: 1471342

24. Kuzmin, I., Botvinkin, A. & Shaimardanov, R. Experimental Rabies and Rabies-Related Infections in Bats. Voprosy Virusologii 39, 17–21 (1994). PMID: 8160441

25. Swanepoel, R. et al. Experimental Inoculation of Plants and Animals with Ebola Virus. Emerging Infectious Diseases (1996). doi.org/10.3201/eid0204.960407

26. Williamson, M. et al. Transmission Studies of Hendra Virus (Equine Morbillivirus) in Fruit Bats, Horses and Cats. Australian Veterinary Journal 76, 813–818 (1998). doi.org/10.1111/j.1751-0813.1998.tb12335.x

27. Sétien, A. et al. Experimental Rabies Infection and Oral Vaccination in Vampire Bats (*Desmodus rotundus*). Vaccine 16, 1122–1126 (1998). doi.org/10.1016/s0264-410x(98)80108-4

28. Williamson, M., Hooper, P., Selleck, P., Westbury, H. & Slocombe, R. Experimental Hendra Virus Infection in Pregnant Guinea-Pigs and Fruit Bats (*Pteropus poliocephalus*). Journal of Comparative Pathology 122, 201–207 (2000). doi.org/10.1053/jcpa.1999.0364

29. Van Der Poel, W. et al. Characterisation of a Recently Isolated Lyssavirus in Frugivorous Zoo Bats. Archives of Virology 145, 1919–1931 (2000). doi.org/10.1007/s007050070066

30. Reid, J. & Jackson, A. Experimental Rabies Virus Infection in *Artibeus jamaicensis* Bats with Cvs-24 Variants. Journal of Neurovirology 7, 511–517 (2001). doi.org/10.1080/135502801753248097

31. Phung, H. et al. Establishment of a Gfp-Based Indicator Cell Line to Quantitate Feline Foamy Virus. Journal of Virological Methods 109, 125–131 (2003). doi.org/10.1016/s0166-0934(03)00062-4

32. Davis, A. et al. Experimental and Natural Infection of North American Bats with West Nile Virus. American Journal of Tropical Medicine and Hygiene 73, 467–469 (2005). doi.org/10.4269/ajtmh.2005.73.467

33. Almeida, M. et al. Experimental Rabies Infection in Haematophagous Bats *Desmodus rotundus*. Epidemiology and Infection 133, 523–527 (2005). doi.org/10.1017/s0950268804003656

34. Aguilar-Setien, A. et al. Salivary Excretion of Rabies Virus By Healthy Vampire Bats. Epidemiology and Infection 133, 517–522 (2005). doi.org/10.1017/s0950268805003705

35. Hughes, G. J. et al. Experimental Infection of Big Brown Bats (*Eptesicus fuscus*) with Eurasian Bat Lyssaviruses Aravan, Khujand, and Irkut Virus. Archives of Virology 151, 2021–2035 (2006). doi.org/10.1007/s00705-005-0785-0

36. Middleton, D. J. et al. Experimental Nipah Virus Infection in Pteropid Bats (*Pteropus poliocephalus*). Journal of Comparative Pathology 136, 266–272 (2007). doi.org/10.1016/j.jcpa.2007.03.002

37. Davis, A. D., Rudd, R. J. & Bowen, R. A. Effects of Aerosolized Rabies Virus Exposure On Bats and Mice. Journal of Infectious Diseases 195, 1144–1150 (2007). doi.org/10.1086/512616

38. Johnson, N. et al. Experimental Study of European Bat Lyssavirus Type-2 Infection in Daubenton’s Bats (*Myotis daubentonii*). J. Gen. Virol. 89, 2662–2672 (2008). doi.org/10.1099/vir.0.2008/003889-0

39. Kuzmin, I. V., Franka, R. & Rupprecht, C. E. Experimental Infection of Big Brown Bats (*Eptesicus fuscus*) with West Caucasian Bat Virus (WCBV) - Towards the Elimination of Rabies in Eurasia. Towards the Elimination of Rabies in Eurasia 131, 327–337 (2008). PMID: 18634495

40. Jackson, F. R. et al. Experimental Rabies Virus Infection of Big Brown Bats (*Eptesicus fuscus*). Journal of Wildlife Diseases 44, 612–621 (2008). doi.org/10.7589/0090-3558-44.3.612

41. Crameri, G. et al. Establishment, Immortalisation and Characterisation of Pteropid Bat Cell Lines. Plos One 4, E8266 (2009). doi.org/10.1371/journal.pone.0008266

42. Jordan, I. et al. Cell Lines from theEgyptian Fruit Bat are Permissive for Modified Vaccinia Ankara. Virus Research 145, 54–62 (2009). doi.org/10.1016/j.virusres.2009.06.007

43. Freuling, C. et al. Experimental Infection of Serotine Bats (*Eptesicus serotinus*) with European Bat Lyssavirus Type 1a. J. Gen. Virol. 90, 2493–2502 (2009). doi.org/10.1099/vir.0.011510-0

44. Kraehling, V. et al. Establishment of Fruit Bat Cells (*Rousettus aegyptiacus*) as a Model System for the Investigation of Filoviral Infection. Plos Neglected Tropical Diseases 4, (2010). doi.org/10.1371/journal.pntd.0000802

45. Watanabe, S. et al. Bat Coronaviruses and Experimental Infection of Bats, the Philippines. Emerging Infect. Dis. 16, 1217–1223 (2010). doi.org/10.3201/eid1608.100208

46. Watanabe, S. et al. Epizootology and Experimental Infection of Yokose Virus in Bats. Comparative Immunology Microbiology and Infectious Diseases 33, 25–36 (2010). doi.org/10.1016/j.cimid.2008.07.008

47. Biesold, S. E. et al. Type I Interferon Reaction to Viral Infection in Interferon-Competent, Immortalized Cell Lines from the African Fruit Bat *Eidolon helvum*. Plos One 6, (2011). doi.org/10.1371/journal.pone.0028131

48. Halpin, K. et al. Pteropid Bats are Confirmed as theReservoir Hosts of Henipaviruses: A Comprehensive Experimental Study of Virus Transmission. The American Journal of Tropical Medicine and Hygiene 85, 946–951 (2011). doi.org/10.4269/ajtmh.2011.10-0567

49. Müller, M. A. et al. Human Coronavirus Emc Does Not Require the SARS-Coronavirus Receptor and Maintains Broad Replicative Capability in Mammalian Cell Lines. Mbio 3, (2012). doi.org/10.1128/mbio.00515-12

50. Paweska, J. T. et al. Virological and Serological Findings in *Rousettus aegyptiacus* Experimentally Inoculated with Vero Cells-Adapted Hogan Strain of Marburg Virus. Plos One 7, E45479 (2012). doi.org/10.1371/journal.pone.0045479

51. Cogswell-Hawkinson, A. et al. Tacaribe Virus Causes Fatal Infection of an Ostensible Reservoir Host, the Jamaican Fruit Bat. J Virol 86, 5791–5799 (2012). doi.org/10.1128/jvi.00201-12

52. Wu, L. et al. Deep Rna Sequencing Reveals Complex Transcriptional Landscape of a Bat Adenovirus. Journal of Virology 87, 503–511 (2013). doi.org/10.1128/jvi.02332-12

53. S, Ekian, V., Lim, D., Prud’homme, P. & Lemay, G. Transient High Level Mammalian Reovirus Replication in a Bat Epithelial Cell Line Occurs without Cytopathic Effect. Virus Research 173, 327–335 (2013). doi.org/10.1016/j.virusres.2013.01.010

54. Davis, A. D., Gordy, P. A. & Bowen, R. A. Unique Characteristics of Bat Rabies Viruses in Big Brown Bats (*Eptesicus fuscus*). Archives of Virology 158, 809–820 (2013). doi.org/10.1007/s00705-012-1551-0

55. Davis, A. D., Jarvis, J. A., Pouliott, C. & Rudd, R. J. Rabies Virus Infection in *Eptesicus fuscus* Bats Born in Captivity (Naive Bats). Plos One 8, (2013). doi.org/10.1371/journal.pone.0064808

56. Ellison, J. A. et al. Multidisciplinary Approach to Epizootiology and Pathogenesis of Bat Rabies Viruses in the United States. Zoonoses and Public Health 60, 46–57 (2013). doi.org/10.1111/zph.12019

57. Perea-Martinez, L. et al. Experimental Infection of *Artibeus intermedius* Bats with Serotype-2 Dengue Virus. Comparative Immunology Microbiology and Infectious Diseases 36, 193–198 (2013). doi.org/10.1016/j.cimid.2012.12.002

58. Davis, A. D., Jarvis, J. A., Pouliott, C. E., Shannon, M. D. M. & Rudd, R. J. Susceptibility and Pathogenesis of Little Brown Bats (*Myotis lucifugus*) to Heterologous and Homologous Rabies Viruses. Journal of Virology 87, 9008–9015 (2013). doi.org/10.1128/jvi.03554-12

59. Krueger, N. et al. Attachment Protein G of an African Bat Henipavirus is Differentially Restricted in Chiropteran and Nonchiropteran Cells. Journal of Virology 88, 11973–11980 (2014). doi.org/10.1128/jvi.01561-14

60. Weis, M. et al. Characterization of African Bat Henipavirus Gh-M74a Glycoproteins. J.Gen.Virol. 95, 539–548 (2014). doi.org/10.1099/vir.0.060632-0

61. Monaghan, P. et al. Detailed Morphological Characterisation of Hendra Virus Infection of Different Cell Types Using Super-Resolution and Conventional Imaging. Virology Journal 11, (2014). doi.org/10.1186/s12985-014-0200-5

62. Eckerle, I. et al. Bat Airway Epithelial Cells: a Novel Tool for the Study of Zoonotic Viruses. Plos One 9, (2014). doi.org/10.1371/journal.pone.0084679

63. Poole, D. S. et al. Influenza a Virus Polymerase is a Site for Adaptive Changes During Experimental Evolution in Bat Cells. Journal of Virology 88, 12572–12585 (2014). doi.org/10.1128/jvi.01857-14

64. Cabrera-Romo, S. et al. Experimental Inoculation of *Artibeus jamaicensis* Bats with Dengue Virus Serotypes 1 or 4 Showed No Evidence of Sustained Replication. American Journal of Tropical Medicine and Hygiene 91, 1227–1234 (2014). doi.org/10.4269/ajtmh.14-0361

65. De Almeida, M. F. et al. Hematologic Profile of Hematophagous *Desmodus rotundus* Bats Before and After Experimental Infection with Rabies Virus. Revista Da Sociedade Brasileira De Medicina Tropical 47, 371–373 (2014). doi.org/10.1590/0037-8682-0169-2013

66. Liang, Y.-Z. et al. Cloning, Expression, and Antiviral Activity of Interferon Β from the Chinese MicroBat, Myotis Davidii. Virologica Sinica 30, 425–432 (2015). doi.org/10.1007/s12250-015-3668-2

67. Glennon, N. B., Jabado, O., Lo, M. K. & Shaw, M. L. Transcriptome Profiling of the Virus-Induced Innate Immune Response in *Pteropus vampyrus* and its Attenuation by Nipah Virus Interferon Antagonist Functions. Journal of Virology 89, 7550–66 (2015). doi.org/10.1128/jvi.00302-15

68. Paweska, J. T. et al. Experimental Inoculation of Egyptian Fruit Bats (*Rousettus aegyptiacus*) with Ebola Virus. Viruses-Basel 8, 29 (2016). doi.org/10.3390/v8020029

69. Jones, M. E. B. et al. Experimental Inoculation of Egyptian Rousette Bats (*Rousettus aegyptiacus*) with Viruses of the Ebolavirus and Marburgvirus Genera. Viruses 7, 3420–3442 (2015). doi.org/10.3390/v7072779

70. Amman, B. R. et al. Oral Shedding of Marburg Virus in Experimentally Infected Egyptian Fruit Bats *(Rousettus aegyptiacus*). Journal of Wildlife Diseases 51, 113–124 (2015). doi.org/10.7589/2014-08-198

71. Moreira, E. A. et al. Synthetically Derived Bat Influenza A-Like Viruses Reveal a Cell Type-But Not Species-Specific Tropism. Proceedings of the National Academy of Sciences of the United States of America 113, 12797–12802 (2016). doi.org/10.1073/pnas.1608821113

72. Hoelzer, M. et al. Differential Transcriptional Responses to Ebola and Marburg Virus Infection in Bat and Human Cells. Scientific Reports 6, (2016). doi.org/10.1038/srep34589

73. Miller, M. R. et al. Broad and Temperature Independent Replication Potential of Filoviruses On Cells Derived from Old and New World Bat Species. Journal of Infectious Diseases 214, S297–S302 (2016). doi.org/10.1093/infdis/jiw199

74. Paweska, J. T. et al. Lack of Marburg Virus Transmission from Experimentally Infected to Susceptible In-Contact Egyptian Fruit Bats. Journal of Infectious Diseases 212, S109–S118 (2015). doi.org/10.1093/infdis/jiv132

75. Bosco-Lauth, A. M., Nemeth, N. M., Kohler, D. J. & Bowen, R. A. Viremia in North American Mammals and Birds After Experimental Infection with Chikungunya Viruses. American Journal of Tropical Medicine and Hygiene 94, 504–506 (2016). doi.org/10.4269/ajtmh.15-0696

76. Zhang, Q. et al. Ifnar2-Dependent Gene Expression Profile Induced By IFN-Α in *Pteropus alecto* Bat Cells and Impact of IFNAR2 Knockout On Virus Infection. Plos One 12, E0182866 (2017). doi.org/10.1371/journal.pone.0182866

77. Kuzmin, I. V. et al. Innate Immune Responses of Bat and Human Cells to Filoviruses: Commonalities and Distinctions. J Virol 91, (2017). doi.org/10.1128/jvi.02471-16

78. Moreira-Soto, A., Soto-Garita, C. & Corrales-Aguilar, E. Neotropical Primary Bat Cell Lines Show Restricted Dengue Virus Replication. Comp. Immunol. Microbiol. Infect. Dis. 50, 101–105 (2017). doi.org/10.1016/j.cimid.2016.12.004

79. Schuh, A. J. et al. Modelling Filovirus Maintenance in Nature By Experimental Transmission of Marburg Virus Between Egyptian Rousette Bats. Nature Communications 8, (2017). doi.org/10.1038/ncomms14446

80. Gerrard, D. L. et al. Transcriptomic Signatures of Tacaribe Virus-Infected Jamaican Fruit Bats. Msphere 2, E00245-17 (2017). doi.org/10.1128/msphere.00245-17

81. Obregón-Morales, C. et al. Experimental Infection of *Artibeus intermedius* with a Vampire Bat Rabies Virus. Comp. Immunol. Microbiol. Infect. Dis. 52, 43–47 (2017). doi.org/10.1016/j.cimid.2017.05.008

82. Arnold, C. E. et al. Transcriptomics Reveal Antiviral Gene Induction in the Egyptian Rousette Bat Is Antagonized in Vitro By Marburg Virus Infection. Viruses 10, (2018). doi.org/10.3390/v10110607

83. Mendez-Ojeda, Lm. et al. Detection of Rabies Virus in Organs Unrelated to theCentral Nervous System of Experimentally-Inoculated Vampire Bats. Revista Mexicana De Ciencias Pecuarias 9, 435–450 (2018). doi.org/10.22319/rmcp.v9i3.4247

84. Suu-Ire, R. et al. Pathogenesis of Bat Rabies in a Natural Reservoir: Comparative Susceptibility of theStraw-Colored Fruit Bat (*Eidolon helvum*) to Three Strains of Lagos Bat Virus. Plos Negl Trop Dis 12, (2018). doi.org/10.1371/journal.pntd.0006311

85. Storm, N., Jansen Van Vuren, P., Markotter, W. & Paweska, J. T. Antibody Responses to Marburg Virus in Egyptian Rousette Bats and Their Role in Protection Against Infection. Viruses 10, (2018). doi.org/10.3390/v10020073

86. Balkema-Buschmann, A. et al. Productive Propagation of Rift Valley Fever Phlebovirus Vaccine Strain Mp-12 in *Rousettus aegyptiacus* Fruit Bats. Viruses-Basel 10, (2018). doi.org/10.3390/v10120681

87. Wong, G. et al. Naturally Occurring Single Mutations in Ebola Virus Observably Impact Infectivity. Journal of Virology 93, (2019). doi.org/10.1128/jvi.01098-18

88. Edenborough, K. M. et al. Dendritic Cells Generated from *Mops condylurus*, a Likely Filovirus Reservoir Host, are Susceptible to and Activated By Zaire Ebolavirus Infection. Front. Immunol. 10, (2019). doi.org/10.3389/fimmu.2019.02414

89. Jones, M. E. B. et al. Clinical, Histopathologic, and Immunohistochemical Characterization of Experimental Marburg Virus Infection in a Natural Reservoir Host, theEgyptian Rousette Bat (*Rousettus aegyptiacus*). Viruses 11, (2019). doi.org/10.3390/v11030214

90. Malmlov, A. et al. Experimental Zika Virus Infection of Jamaican Fruit Bats (*Artibeus jamaicensis*) and Possible Entry of Virus Into Brain Via Activated Microglial Cells. Plos Neglected Tropical Diseases 13, (2019). doi.org/10.1371/journal.pntd.0007071

91. Ciminski, K. et al. Bat Influenza Viruses Transmit Among Bats But are Poorly Adapted to Non-Bat Species. Nature Microbiology 4, 2298–2309 (2019). doi.org/10.1038/s41564-019-0556-9

92. Bokelmann, M. et al. Utility of Primary Cells to Examine Npc1 Receptor Expression in *Mops condylurus*, a Potential Ebola Virus Reservoir. Plos Negl Trop Dis 14, E0007952 (2020). doi.org/10.1371/journal.pntd.0007952

93. Irving, A. T. et al. Interferon Regulatory Factors Irf1 and Irf7 Directly Regulate Gene Expression in Bats in Response to Viral Infection. Cell Reports 33, (2020). doi.org/10.1016/j.celrep.2020.108345

94. Chen, M., Tachedjian, M., Marsh, G. A., Cui, J. & Wang, L.-F. Distinct Cell Transcriptomic Landscapes Upon Henipavirus Infections. Frontiers in Microbiology 11, (2020). doi.org/10.3389/fmicb.2020.00986

95. Seifert, S. N. et al. *Rousettus aegyptiacus* Bats Do Not Support Productive Nipah Virus Replication. J Infect Dis 221, S407–S413 (2020). doi.org/10.1093/infdis/jiz429

96. Amman, B. R. et al. Experimental Infection of Egyptian Rousette Bats (*Rousettus aegyptiacus*) with Sosuga Virus Demonstrates Potential Transmission Routes for a Bat-Borne Human Pathogenic Paramyxovirus. Plos Neglect. Trop. Dis. 14, E0008092 (2020). doi.org/10.1371/journal.pntd.0008092

97. Begeman, L. et al. Experimental Lagos Bat Virus Infection in Straw-Colored Fruit Bats: a Suitable Model for Bat Rabies in a Natural Reservoir Species. Plos Neglected Tropical Diseases 14, (2020). doi.org/10.1371/journal.pntd.0008898

98. Gorka, M. et al. Characterization of Experimental Oro-Nasal Inoculation of Seba’s Short-Tailed Bats (*Carollia perspicillata*) with Bat Influenza a Virus H18n11. Viruses 12, (2020). doi.org/10.3390/v12020232

99. Banerjee, A. et al. Selection of Viral Variants During Persistent Infection of Insectivorous Bat Cells with Middle East Respiratory Syndrome Coronavirus. Scientific Reports 10, (2020). doi.org/10.1038/s41598-020-64264-1

100. Tarigan, R. et al. Role of Pattern Recognition Receptors and Interferon-Beta in Protecting Bat Cell Lines from Encephalomyocarditis Virus and Japanese Encephalitis Virus Infection. Biochemical and Biophysical Research Communications 527, 1–7 (2020). doi.org/10.1016/j.bbrc.2020.04.060

101. Woon, A. P. et al. Acute Experimental Infection of Bats and Ferrets with Hendra Virus: Insights Into theEarly Host Response of theReservoir Host and Susceptible Model Species. Plos Pathog. 16, E1008412 (2020). doi.org/10.1371/journal.ppat.1008412

102. Schlottau, K. et al. SARS-Cov-2 in Fruit Bats, Ferrets, Pigs, and Chickens: An Experimental Transmission Study. The Lancet Microbe 1, E218–E225 (2020). doi.org/10.1016/s2666-5247(20)30089-6

103. Kuzmin, I. V., Ramanathan, P., Basler, C. F. & Bukreyev, A. Effects of Overexpression of theEgyptian Fruit Bat Innate Immune Genes On Filovirus Infections in theHost Cells. Frontiers in Virology 1, (2021). doi.org/10.3389/fviro.2021.759655

104. Gultom, M. et al. Susceptibility of Well Differentiated Airway Epithelial Cell Cultures from Domestic and Wild Animals to Severe Acute Respiratory Syndrome Coronavirus 2. Emerging Infectious Diseases 27, 1811–1820 (2021). doi.org/10.3201/eid2707.204660

105. Elbadawy, M. et al. Establishment of Intestinal Organoid from *Rousettus leschenaultii* and theSusceptibility to Bat-associated Viruses, SARS-Cov-2 and Pteropine Orthoreovirus. International Journal of Molecular Sciences 22, (2021). doi.org/10.3390/ijms221910763

106. Aurine, N. et al. Reprogrammed Pteropus Bat Stem Cells as a Model to Study Host-Pathogen Interaction During Henipavirus Infection. Microorganisms 9, (2021). doi.org/10.3390/microorganisms9122567

107. Neill, W. A. & Kading, R. C. Viral Ecology and Natural Infection Dynamics of Kaeng Khoi Virus in Cave-Dwelling Wrinkle-Lipped Free-Tailed Bats (*Chaerephon plicatus*) in Thailand. Diseases 9, (2021). doi.org/10.3390/diseases9040073

108. Hall, J. S. et al. Experimental Challenge of a North American Bat Species, Big Brown Bat (*Eptesicus fuscus*), with SARS-Cov-2. Transbound Emerg Dis 68, 3443–3452 (2021). doi.org/10.1111/tbed.13949

109. Halwe, N. J. et al. Egyptian Fruit Bats (*Rousettus aegyptiacus*) were Resistant to Experimental Inoculation with Avian-Origin Influenza a Virus of Subtype H9N2, But are Susceptible to Experimental Infection with Bat-Borne H9N2 Virus. Viruses-Basel 13, (2021). doi.org/10.3390/v13040672

110. Fagre, A. C. et al. Subgenomic Flavivirus Rna (Sfrna) associated with Asian Lineage Zika Virus Identified in Three Species of Ugandan Bats (Family Pteropodidae). Scientific Reports 11, 8370 (2021). doi.org/10.1038/s41598-021-87816-5

111. Papies, J. et al. Reduced IFN-ß Inhibitory Activity of Lagos Bat Virus Phosphoproteins in Human Compared to *Eidolon helvum* Bat Cells. Plos One 17, (2022). doi.org/10.1371/journal.pone.0264450

112. Kirejczyk, S. G. M. et al. Histopathologic and Immunohistochemical Evaluation of Induced Lesions, Tissue Tropism and Host Responses Following Experimental Infection of Egyptian Rousette Bats (*Rousettus aegyptiacus*) with theZoonotic Paramyxovirus, Sosuga Virus. Viruses-Basel 14, 1278 (2022). doi.org/10.3390/v14061278

113. Bosco-Lauth, A. M. et al. Experimental Infection of Brazilian Free-Tailed Bats (*Tadarida brasiliensis*) with Two Strains of SARS-Cov-2. Viruses 14, 1809 (2022). doi.org/10.3390/v14081809

114. Gamage, A. M. et al. Single-Cell Transcriptome Analysis of the in Vivo Response to Viral Infection in theCave Nectar Bat *Eonycteris spelaea*. Immunity 55, 2187-2205.E5 (2022). doi.org/10.1016/j.immuni.2022.10.008

115. Ohkura, S. et al. Characterization of Megabat-Favored, Ca-Dependent Susceptibility to Retrovirus Infection. Journal of Virology 97, (2023). doi.org/10.1128/jvi.01803-22

116. Toth, G. E. et al. Isolation and Genome Characterization of Lloviu Virus from Italian Schreibers’s Bats. Scientific Reports 13, (2023). doi.org/10.1038/s41598-023-38364-7

117. Su, A. et al. Infection Studies with Airway Organoids from *Carollia perspicillata* Indicate That the Respiratory Epithelium Is Not a Barrier for Interspecies Transmission of Influenza Viruses. Microbiology Spectrum 11, (2023). doi.org/10.1128/spectrum.03098-22

118. Mah, M. G. et al. Spike-Independent Infection of Human Coronavirus 229e in Bat Cells. Microbiology Spectrum 11, (2023). doi.org/10.1128/spectrum.03483-22

119. Harazim, M. et al. Transcriptomic Responses of Bat Cells to European Bat Lyssavirus 1 Infection Under Conditions Simulating Euthermia and Hibernation. Bmc Immunology 24, (2023). doi.org/10.1186/s12865-023-00542-7

120. Hashimi, M. et al. Antiviral Responses in a Jamaican Fruit Bat Intestinal Organoid Model of SARS-Cov-2 Infection. Nature Communications 14, (2023). doi.org/10.1038/s41467-023-42610-x

121. Ahn, M. et al. Bat ASC2 Suppresses Inflammasomes and Ameliorates Inflammatory Diseases. Cell 186, 2144+ (2023). doi.org/10.1016/j.cell.2023.03.036

122. Fu, F. et al. Bat Sting Drives Ifn-Beta Production in Anti-Rna Virus Innate Immune Response. Front. Microbiol. 14, (2023). doi.org/10.3389/fmicb.2023.1232314

123. Chan, L. L. Y. et al. Generation of Self-Replicating Airway Organoids from theCave Nectar Bat *Eonycteris spelaea* as a Model System for Studying Host-Pathogen Interactions in theBat Airway Epithelium. Emerging Microbes & Infections 12, (2023). doi.org/10.1080/22221751.2022.2148561

124. Aguilar-Setien, A. et al. Experimental Infection of *Artibeus lituratus* Bats and No Detection of Zika Virus in Neotropical Bats from French Guiana, Peru, and Costa Rica Suggests a Limited Role of Bats in Zika Transmission. Plos Neglected Tropical Diseases 17, (2023). doi.org/10.1371/journal.pntd.0010439

125. Hall, J. S. et al. Experimental Infection of Mexican Free-Tailed Bats (*Tadarida brasiliensis*) with SARS-Cov-2. Msphere 8, E00263-22 (2023). doi.org/10.1128/msphere.00263-22

126. Burke, B. et al. Regulatory T Cell-Like Response to SARS-Cov-2 in Jamaican Fruit Bats (*Artibeus jamaicensis*) Transduced with Human ACE2. Plos Pathogens 19, (2023). doi.org/10.1371/journal.ppat.1011728

127. Guito, J. C. et al. Peripheral Immune Responses to Filoviruses in a Reservoir Versus Spillover Hosts Reveal Transcriptional Correlates of Disease. Frontiers in Immunology 14, (2024). doi.org/10.3389/fimmu.2023.1306501
